# Supplementary material for: Content-rich biological network constructed by mining PubMed abstracts
Source: BMC Bioinformatics. 2004 Oct 8;5:147. doi: 10.1186/1471-2105-5-147 (PMC528731; doi:10.1186/1471-2105-5-147)
Supplement: Additional File 2 — The original results of the above study (non-essential files are deleted to keep the file size under the limit set by BMC bioinformatics). [file 1471-2105-5-147-S2.bz2 › chilibotAdditionalFile2/dip30/352ID7834747E4685/html/BCL2_BAG1.html]

 


 **BCL2** and **BAG1** 
  
Found 100 abstracts in PubMed, retrieved 30.  
 

 What does Google say? 
 PDF only 
| .edu only 

---

**Interactive relationship** (e.g. stimulation, inhibition, etc)

**Neutral relationship**- BAG 1  [ **BAG1** ]  is a recently identified Bcl 2  [ **BCL2** ]  interacting anti apoptotic protein.  Ref: 12402153 Br J Cancer, 2002
- Bag 1  [ **BAG1** ]  is a recently identified bcl 2  [ **BCL2** ]  related protein and is known to be linked to the biological aggressiveness of some carcinomas.  Ref: 12680148 Anticancer Res< MedlineTA>Anticancer Res, 2002
- Bcl 2  [ **BCL2** ]  associated athanogene BAG  [ **BAG1** ]  family proteins are BAG  [ **BAG1** ]  domain containing proteins that interact with the heat shock proteins 70, both constitutive Hsc70 and inducible Hsp70.  Ref: 12406544 Cancer Lett, 2002
- Bag 1  [ **BAG1** ]  exerts powerful antiapoptotic effects by binding and stabilizing Bcl 2  [ **BCL2** ]  and interacting with the tumor necrosis factor receptor type I induced death signal.  Ref: 12215270 Hum Gene Ther, 2002
- Here, we found that honokiol induced apoptotic cell death was accompanied by upregulation of Bad and downregulation of Bcl XL, while honokiol had no effect on the levels of Bcl 2  [ **BCL2** ] , Bcl XS, Bag 1  [ **BAG1** ] , Bax and Bak proteins.  Ref: 12007567 Biochem Pharmacol, 2002
- It was revealed that LIGHT treatment resulted in down regulation of anti apoptosis Bcl 2  [ **BCL2** ]  family member Bcl 2  [ **BCL2** ] , Bcl X L, Bag 1  [ **BAG1** ] , and Mcl 1.  Ref: 12767529 Cancer Lett, 2003
- Of particular relevance to tumour cells, BAG 1  [ **BAG1** ]  interacts with the anti apoptotic BCL 2  [ **BCL2** ]  protein, various nuclear hormone receptors and the 70 kDa heat shock proteins, Hsc70 and Hsp70.  Ref: 12618309 Biochim Biophys Acta, 2003
- The predictive value of bcl 2  [ **BCL2** ] , bax, bcl xL, bag 1  [ **BAG1** ] , fas, and fasL for chemotherapy response in advanced breast cancer.  Ref: 11895913 Clin Cancer Res, 2002
- BAG 1  [ **BAG1** ]  was originally identified as a binding partner of anti apoptotic factor Bcl 2  [ **BCL2** ]  Takayama et al.  Ref: 12565851 Biochem Biophys Res Commun, 2003
- gossypol induced apoptosis triggers down regulation of anti apoptosis Bcl 2  [ **BCL2** ]  members Bcl X L, Bag 1  [ **BAG1** ]  and Mcl 1, up regulation of pro apoptosis Bcl 2  [ **BCL2** ]  member Bak, activation of caspase,, and 9, up regulation of Apaf 1, release of cytochrome c cyto c from mitochondria, and activation of both DFF45 and PARP.  Ref: 12818369 Biochem Pharmacol, 2003
- Both BAG 1  [ **BAG1** ]  and BAG  [ **BAG1** ]  3 CAIR 1 interact with Bcl 2  [ **BCL2** ]  and have been shown to have a supra additive anti apoptotic effect with Bcl 2  [ **BCL2** ] .  Ref: 12406544 Cancer Lett, 2002
- The anti apoptotic activities of BAG  [ **BAG1** ]  family proteins may be dependent on their interactions with Hsc70 Hsp70 and or binding to Bcl 2  [ **BCL2** ] .  Ref: 12406544 Cancer Lett, 2002
- BAG 1  [ **BAG1** ]  BCL 2  [ **BCL2** ]  athanogene 1, a multifunctional protein which associates with steroid hormone receptors including the oestrogen receptor and the anti apoptotic BCL 2  [ **BCL2** ]  protein, regulates steroid hormone dependent transcription and apoptosis.  Ref: 12081204 J Pathol, 2002
- CONCLUSION These findings suggest that bag 1  [ **BAG1** ]  may interact with bcl 2  [ **BCL2** ]  to play an important role in thyroid neoplasm before anaplastic transformation, and the disruption of events mediated by bag 1  [ **BAG1** ]  and bcl 2  [ **BCL2** ]  may be a typical characteristic of undifferentiated carcinoma.  Ref: 12680148 Anticancer Res< MedlineTA>Anticancer Res, 2002
- The clonogenic growth of breast cancer cell lines was inhibited by a ligand for PPARgamma troglitazone, TGZ combined with a ligand for either retinoid X receptor RXR LG10069 4 8 cell lines, RAR ATRA 5 8 cell lines or RAR RXR and RXR RXR 9 cis RA 5 8 cell lines independent of their expression of bcl 2  [ **BCL2** ] , bag 1  [ **BAG1** ] , ERalpha, and p53.  Ref: 12186376 Breast Cancer Res Treat, 2002
- In 126 patients, histological blocks of primary tumors were available for immunohistochemical analysis of bax, bcl 2  [ **BCL2** ] , bcl xL, bag 1  [ **BAG1** ] , fas and fasL.  Ref: 11895913 Clin Cancer Res, 2002
- While Bcl 2  [ **BCL2** ]  levels were permanently and Bag 1  [ **BAG1** ]  levels transiently elevated upon therapy, other apoptosis regulatory genes revealed no alterations.  Ref: 12667663 J Neuroimmunol, 2003
- Bis also called Bag  [ **BAG1** ]  3, identified as a novel Bcl 2  [ **BCL2** ]  interacting protein, has been shown to enhance anti cell death activity of Bcl 2  [ **BCL2** ] .  Ref: 12061864 Exp Neurol, 2002

**Non-interactive relationship** (e.g. studied together, co-existance, homology, etc.)

- Bag 1  [ **BAG1** ]  expression in thyroid neoplasm its correlation with Bcl 2  [ **BCL2** ]  expression and carcinoma dedifferentiation.  Ref: 12680148 Anticancer Res< MedlineTA>Anticancer Res, 2002
- The present study was performed to clarify the clinical implications of BAG 1  [ **BAG1** ] , a bcl 2  [ **BCL2** ]  binding protein in esophageal squamous cell carcinoma ESCC.  Ref: 12823208 Dis Esophagus, 2003
- These in vitro results suggest that distinct isoforms of BAG 1  [ **BAG1** ]  have different anti apoptotic functions and their functions may be correlated to increased Bcl 2  [ **BCL2** ]  expression.  Ref: 12370827 Oncogene, 2002
- Intragraft expression of antiapoptotic Bcl 2  [ **BCL2** ]  and Bag 1  [ **BAG1** ]  was increased in Ad HO 1 treated orthotopic liver transplants, as compared with Ad beta Gal controls.  Ref: 12134106 Transplantation, 2002
- BAG  [ **BAG1** ]  Bcl 2  [ **BCL2** ]  associated athanogene proteins are molecular chaperone regulators that affect diverse cellular pathways.  Ref: 12058034 J Biol Chem, 2002
- Four anti apoptotic proteins were altered 2.1 to 14.6 fold for Bcl 2  [ **BCL2** ]  and BAG 1  [ **BAG1** ]  isoform p33 and p29.  Ref: 12706450 Eur J Pharmacol, 2003
- The hsp70 hsc70 associating protein Hap46 of human origin, also called BAG  [ **BAG1** ]  1M Bcl 2  [ **BCL2** ]  associated athanogene 1, has been characterized previously as a DNA binding protein, which is able to stimulate transcription.  Ref: 12682371 Nucleic Acids Res, 2003
- This screen revealed the elevated expression of BCL 2  [ **BCL2** ] , BCL 2A1, BAG 1  [ **BAG1** ]  and TACE upon stimulation with GM CSF.  Ref: 12648514 Leuk Res, 2003
- Peripheral immune cells were investigated for apoptosis and for the expression of apoptosis regulatory genes CD95, CD95 ligand, FLIP, Bcl 2  [ **BCL2** ] , Bcl X L, Bag 1  [ **BAG1** ] , and caspase 3 by quantitative real time PCR.  Ref: 12667663 J Neuroimmunol, 2003
- RESULTS Of the investigated factors, bag 1  [ **BAG1** ]  correlated positively with bax, bcl 2  [ **BCL2** ] , and fasL, and fasL correlated positively with fas and bax.  Ref: 11895913 Clin Cancer Res, 2002
- To determine whether changes in the expression of proteinases, 73 kDa constitutive heat shock cognate protein Hsc70 and stress inducible 72 kDa heat shock protein Hsp70 Hsc Hsp70, and Bcl 2  [ **BCL2** ]  associated gene product 1 BAG 1  [ **BAG1** ]  contribute to the remodeling response of muscle tissue, tibialis anterior muscles of male Sprague Dawley rats were injected with 0.75% bupivacaine and removed at, 1, or 35 days postinjection n = 5 7 group.  Ref: 12791605 Am J Physiol Endocrinol Metab, 2003
- ... other Bcl 2  [ **BCL2** ]  family proteins including Bax, Bcl 2  [ **BCL2** ] , Bcl XL, and Bag  [ **BAG1** ]  remained unchanged.  Ref: 12874837 J Cell Biochem, 2003
- MATERIALS AND METHODS We immunohistochemically investigated bag 1  [ **BAG1** ]  and bcl 2  [ **BCL2** ]  expression in various thyroid neoplasms using monoclonal antibodies.  Ref: 12680148 Anticancer Res< MedlineTA>Anticancer Res, 2003
- These data implicate induction of the stress response and its modulation by the chaperone cofactor and Bcl 2  [ **BCL2** ]  family member BAG 1  [ **BAG1** ] , after TBI in humans.  Ref: 12558971 J Neurochem, 2003
- Down regulation of Bcl 2  [ **BCL2** ]  interacting protein BAG 1  [ **BAG1** ]  confers resistance to anti cancer drugs.  Ref: 12565851 Biochem Biophys Res Commun, 2003
- The expression of Bis also called Bag  [ **BAG1** ]  3, a Bcl 2  [ **BCL2** ]  binding protein, was investigated in the rat kainic acid KA model of temporal lobe epilepsy.  Ref: 12085992 Exp Mol Med, 2002
- Our data demonstrated that Gyp induced apoptotic cell death was accompanied by up regulation of Bax, Bak and Bcl X, and down regulation of Bcl 2  [ **BCL2** ]  and Bad, while it had no effect on the level of Bag 1  [ **BAG1** ]  protein.  Ref: 12065092 Cancer Lett, 2002
